# The PEG-PCL-PEG Hydrogel as an Implanted Ophthalmic Delivery System after Glaucoma Filtration Surgery; a Pilot Study

**Published:** 2014

**Authors:** Ribo Peng, Gang Qin, Xiabin Li, Hongbin Lv, Zhiyong Qian, Ling Yu

**Affiliations:** 1 Department of Ophthalmology,; 2 Department of Otorhinolaryngology,; 3 Department of Pathology, the Affiliated Hospital of Luzhou Medical College, Luzhou of Sichuan Province, 646000, China,; 4 State Key Laboratory of Biotherapy and Cancer Center , West China Hospital , Sichuan University, Chengdu , Sichuan Province 610041, China

**Keywords:** PEG-PCL-PEG, PECE Hydrogel, Ophthalmic Delivery System, Glaucoma Filtration Surgery, Filtration Fistula

## Abstract

Currently, filtration surgery has been considered as the most effective therapy for glaucoma; however, the scar formation in the surgical area may often lead to failure to the procedure. An implanted drug delivery system may provide localized and sustained release of a drug over an extended period. Poly (ethylene glycol)-poly (ε-caprolactone)-poly (ethylene glycol) (PEG-PCL-PEG, PECE) hydrogel has been successfully synthesized and determined as thermosensitive and biocompatible. In order to overcome the limitations of common local ophthalmic medications, we investigated the function of a self-assembled PECE hydrogel as an intracameral injection-implanted drug carrier to inhibit the formation of postoperative scarring. Following bevacizumal-loaded hydrogel intracameral injection into rabbit eyes, the status of the bleb and filtration fistula formed following the filtering surgery were examined through pathologic evaluation. Due to the sustained release of bevacizumab from the hydrogel, neovascularization and scar formation were inhibited; moreover, there were no corneal abnormalities and other ocular tissue damage found in the rabbits. This suggests that the PECE hydrogel may be considered as the novel biomaterial with potential as a sustained release system in glaucoma filtering surgery. Further studies require in shedding the light on the subject.

## INTRODUCTION

Glaucoma is one of the major causes of irreversible blindness in the world and is usually associated with increased intraocular pressure (IOP) (1,2). Glaucoma filtration surgery is performed by creating a fistula between the subconjunctival space and the anterior chamber to allow the drainage of aqueous humor into the filtering bleb, resulting in lowering the IOP (3). Modulation of wound healing is critical to ensure successful surgery. Unfortunately, glaucoma filtration surgery fails in 30–50% of patients because of fibroblast proliferation and collagen deposition at the site of the filtration bleb, preventing further drainage of aqueous humor (4). There are many ophthalmic drug delivery systems that can be used following filtration surgery. Conventional delivery systems such as eye drops, ointments, or suspensions are the most preferred routes of administration to the anterior segment of the eye. The bioavailability of topical instillation of drugs is, however, usually low since the absorption of eye drops is severely limited by the relatively impermeable cornea. Additionally, multiple subconjunctival and intracameral injections of antiscarring drugs following glaucoma filtering surgery are often required to maintain a high drug concentration (5). Moreover, not only are the frequent injections inconvenient and uncomfortable for the patient, but these agents can also cause cell apoptosis and death. This injection process is usually associated with several undesirable complications such as corneal epithelial defects, cataracts, endophthalmitis and other ocular diseases (6). Frequent instillation of concentrated solutions is required to achieve the desired therapeutic effects. There are few satisfactory treatments due to the lack of an ideal drug delivery system. We may need a novel and promising ophthalmic drug delivery system for these diseases. As it is a new biomaterial, a biodegradable poly (PEG-PCL-PEG, PECE) triblock copolymer was successfully synthesized, resulting in a flowing solution at low temperatures that turned into a nonflowing gel at body temperature (1). The PECE hydrogel applied through intracameral injection could be a novel vector for an ophthalmic drug delivery system and gene therapy. Bevacizumab (Avastin), a synthetic monoclonal antibody used against vascular endothelial growth factor (anti-VEGF), was reported to limit scar formation following glaucoma filtration surgery and to effectively decrease patients’ IOP (7,8). Several reports have described the successful, off-label intravitreal use of bevacizumab for treatment of neovascularization associated with proliferative diabetic retinopathy, age-related macular degeneration, and neovascular glaucoma (9,10). Furthermore, bevacizumab may be a beneficial agent for limiting scar tissue formation and thereby improving success following filtration surgery (11).

**Is the hydrogel an ideal drug delivery system?**

It is obvious that the inhibition of scar tissue formation is limited while the bevacizumab is directly administrated through intracameral injection and subsequently rapidly eliminated from the body. Therefore, a suitable container allowing sustained release of bevacizumab is needed. In clinical application, ocular anterior segment implanted biomaterials should ideally meet the following qualities: (a) the implanted materials should be injectable and the process of implantation simple, moreover, they should have certain viscoelasticity and keep flowing in the ocular anterior segment; (b) the implanted materials should be non-immunogenic to not cause inflammation and foreign body adverse reactions; (c) the implanted materials should completely degrade over a certain time and the materials from the degradation process can be an effectively controlled drug release into the anterior chamber to cure ocular diseases.

 To date, various drug delivery devices such as biodegradable amphiphilic block copolymers have been synthesized and applied as drug delivery systems (12-14). For local sustainable release of therapeutic agents, hydrogels are the preferred choice due to their good biodegradability, biocompatibility, huge drug capacity and response to the physical/chemical stimulus (15,16). Hydrogels have been studied extensively in various applications such as medical devices and drug delivery system (17,18). The gelation of hydrogel in the body may lower the rate of diffusion of the entrapped drug following being loaded with therapeutic agents, which enhances the drug retention and bioavailability (19). Furthermore, injectable hydrogels would be in a soluble form prior administration in the body, however once administered, undergo gelation in situ to form a gel. The porous structure of hydrogels makes them perfect carriers for therapeutic macromolecules, such as protein, RNA or DNA. Hydrogel can also be used as a nonviral gene delivery vehicle. It is reported that supercoiled pDNA entrapped in hydrogel expressed maximally at one day and lasted for three days (20,21). For the macrostructure of bevacizumab, the hydrogel systems are suitable carriers for its sustainable delivery. Additionally, because of its thermoresponsive characteristic, the hydrogel can be injected in a liquid form to the juxtascleral region or the vitreous cavity via a small-gauge needle. This system is designed to optimize the antiangiogenic effects and minimize the potential ectopic effects of a large bolus delivery (22). Thermosensitive hydrogels therefore have great potential in the topical delivery of bevacizumab.

 Recently, a series of block copolymers consisting of PEG and PCL or PLA, which can form micelles by self-assembling, or thermosensitive hydrogels have been widely reported (23,24). Based on the PEG as the hydrophilic segment, and PCL or PLA as hydrophobic segment, different kinds of hydrogels can be formed that include PCEC hydrogel, PECE hydrogel, PLA-PEG-PLA hydrogel and PEG-PLA hydrogel. Among these, PECE hydrogel contains the basic components of PEG and PCL, which are biocompatible and have been used in several Food and Drug Administration-approved products. For instance, the in vivo gel formation and degradation behavior were noted by subcutaneously injecting aqueous PECE solution into mice. The PECE hydrogel was also an effective, safe, and convenient agent for preventing post-surgical intra-abdominal adhesions, and was proved to be safe in BALB/c mice in vivo by acute toxicity testing (25,26). Consequently, the PECE hydrogel is believed to be promising for in situ gel-forming controlled drug delivery systems that are injectable flowing solutions at low temperature but that turn into gel at body temperature in vivo (27). The good sustained release properties of hydrogels have been confirmed in vitro. The sol–gel–sol transition temperature range could be varied, and the prepared PECE hydrogels have proved to be thermosensitive, biocompatible, and bioabsorbable (28). With further consideration of the loading and releasing properties of PECE hydrogel to small molecules, such as HK, curcumin, DOX, cisplatin and so forth, it is rational to predict that this hydrogel will exhibit excellent performance in the topical delivery of bevacizumab. We have hypothesized that it could be delivered in a liquid form, which is much more convenient and also temperature-sensitive, then it could form a gel in situ and release drugs continuously after intraocular injection or instillation. Furthermore, ocular tissues could absorb the PECE hydrogel completely without any permanent damage. PECE hydrogel may be an implanted drug delivery system for the inhibition of postoperative scarring formation. 

## METHODS

The PECE hydrogel has great biocompatibility, biodegradability, and sustained release properties in the eye and it is hoped it will be a creative and promising ophthalmic drug delivery system in the future. So we speculate that PECE hydrogel through intracameral injection is as a novel potential, in situ, sustained ophthalmic drug delivery system that can be applied to prevent scarring formation after trabeculectomy. However, the toxicity of PECE hydrogel was concentration-dependent according to our pilot study with different concentrations (5 wt %, 10 wt %, 15 wt % and 20 wt %). Endothelia of rabbits were treated with 5 wt %, 10 wt% and 15 wt% PECE hydrogel seemed regular. However, 5 wt% and 10 wt% PECE hydrogel were not conducive to sustained release, because they were degraded more early than 15 wt% PECE hydrogel. In addition, 20 wt% PECE hydrogel could result in endothelial injury more severely than 15 wt% PECE hydrogel. We eventually found that the slight toxicity of 15 wt% PECE hydrogel is reasonable. 

 PECE triblock copolymer was dissolved in a balanced salt solution (BSS) at a designated temperature and at the concentration of 15 wt% to form PECE hydrogels; these PECE hydrogels were then kept at 4° C before being used (28). In the self-assembly process hydrogel can be directly loaded with appropriate drugs. We loaded bevacizumab into the hydrogel, building a bevacizumab controlled-release system, and applied this kind of controlled-release system following filtration surgery through intracameral injections into rabbit eyes. Bleb survival and characteristics were evaluated over a 28-day period. The rabbits were sacrificed with an overdose of pentobarbital on the 28th day. Histology of the surgical eyes was performed to evaluate and grade the amount of scarring and fibrosis in each group.

## RESULTS

**Figure 1:**
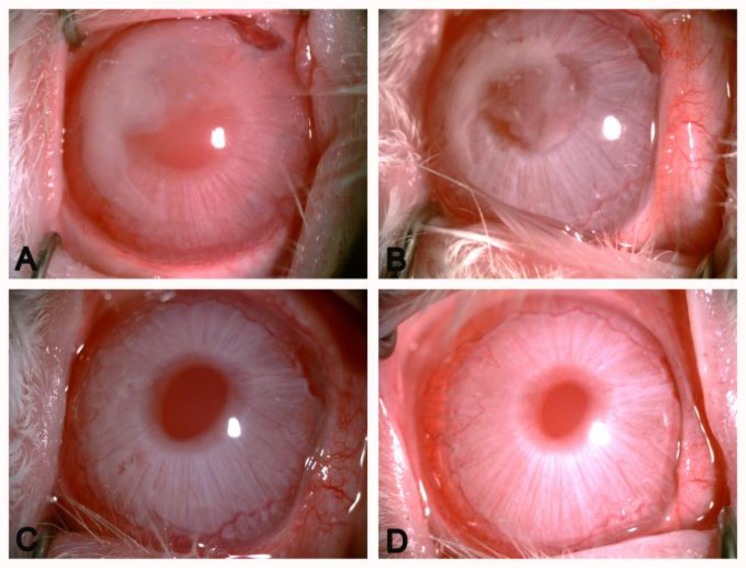
In vivo gel formation of PECE hydrogel in the rabbit anterior chamber. PECE was absorbed completely within 3 weeks. A: 1d after injection. B: 7d after injection. C: 14d after injection D: 21d after injection (× 40 magnification).

**Figure 2:**
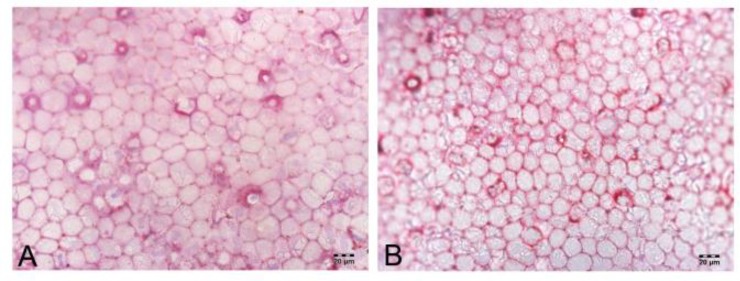
Combined staining of rabbit endothelia with Typan blue and alizarin red after PECE injection into anterior chamber. Staining of intercellular borders with alizarin red showed the mosaic pattern of normal cells and low proportion of dead cells. A: control eye without treatment B: PECE-bevacizumab injection eye (×400 magnification)

The rabbits’ eyes were examined carefully to detect the toxic effect of PECE hydrogel on ocular tissues and to evaluate the biocompatibility of novel hydrogel following injection into the rabbits’ anterior chambers. In the whole experimental process, the rabbits’ anterior chambers that were injected PECE hydrogels did not show any corneal abnormalities, cataract, inflammatory signs, or other ocular disorders by slit-lamp biomicroscopy and indirect ophthalmoscopy. Bevacizumab could be administered slowly, smoothly and efficiently by release from a hydrogel controlled-release system. In the anterior chamber, PECE hydrogel on the corneal endothelia burst into fragments on contact with the aqueous humor because of hydrolysis. Hydrogel was absorbed completely within 3 weeks (Figure 1). These phenomena indicated water gel injected in rabbit eyes with good biocompatibility. We also tested the high IOP of eyes that had been injected with bevacizumab-loaded hydrogel and found that the anterior chamber injected bevacizumab-loaded hydrogel maintained relatively lower IOP, compared with the Bevacizumab group and BSS group following trabeculectomy (Table 1). We speculated that following a bevacizumab-loaded hydrogel injection into the anterior chamber of rabbits, the main degradation products, as a single amino acid, had non-toxic side effects on the trabecular meshwork cells in rabbit eyes. In addition, there was a period of time for hydrogel degradation, so PECE hydrogel released bevacizumab constantly to encourage antiscar formation and control IOP in rabbit eyes. We will evaluate this hypothesis in the future since this study has been performed as a pilot report. Finally, the pathological tissue slices of the injected eyes, including the cornea, lens, iris, ciliary body and retina and found there were no tissue anomalies. The structure of each layer of the above tissues showed no infiltration by inflammatory cells or obvious irritations to rabbit eyes (data not shown). Dual staining of corneal endothelium with Typan blue and Alizarin red was also performed. The rabbits’ endothelial cells were nearly a regular hexagon shape and intact following the bevacizumab-loaded hydrogel intracameral injection (Figure 2). Repeated measures ANOVA was analyzed (F=10.135, *P*=0.012), suggesting that the difference between the three groups was statistically significant. Then we found there was no significant difference between BSS group and Bevacizumab group, while a significant decrease in IOP was found in the Bevacizumab-loaded hydrogel group compared with the BSS group or Bevacizumab group (*P* <0.01 and *P* <0.05, respectively) (n=3 rabbit in each group).

**Table 1:** Preoprative and postoperative intraocular pressures following the filtration surgery (mmHg).

**Group**	**Preoperative IOP**	**Postoperative IOP**					
		**1(d)**	**3(d)**	**7(d)**	**14(d)**	**21(d)**	**28(d)**
**BSS Group**	16.997±0.335	14.773±0.836	15.000±0.000	14.773±0.510	14.887±0.196	15.443±0.510	15.220±0.191
**Bevacizumab Group**	17.220±0.381	15.443±0.510	15.443±0.510	14.997±0.335	14.110±0.191	13.553±0.387	13.777±0.387
**Bevacizumab-loaded Hydrogel Group**	16.667±0.557	15.553±0.508	14.887±0.196	14.220±0.191	13.443±0.510	13.110±0.840	12.997±0.335

## DISSCUSSION

To assess whether these PECE hydrogel applications through intracameral injection can be a sustained and secure ophthalmic drug delivery system in clinical practice, further studies on toxicity evaluation could be conducted. More evidence on the ocular drug delivery nanocarriers with antiscarring drugs following glaucoma filtration surgery will support our hypothesis. Hydrophilic agents can easily mix with hydrogel and form sustained release medications. Intracameral injection of hydrogel at relatively low concentration has little effect on cell viability, IOP and histopathology of ocular tissues in toxicity evaluation (28). This is especially useful for antiscarring treatment after glaucoma filtration surgery in clinical practice. Although, there are many anatomical differences between rabbit and human eyes (29), the overall pattern of wound healing is similar to scars found in humans (30). As a result, intracameral injection of drug-loaded hydrogel could avoid the need for frequent subconjunctival injections and decrease the toxic ocular side effects caused by intraoperative topical applications. This approach should be further investigated.

## CONCLUSION

The results indicated that PECE hydrogel is of good biodegradability and biocompatibility when injected into the anterior chamber of rabbits. The drug-loaded hydrogels provide a great opportunity to increase the therapeutic efficacy of glaucoma filtration surgery. This technique not only avoids frequent subconjunctival or intracameral injection of drugs given thereby significantly reducing the side effects of drugs on the surrounding normal tissue, but can also control the filtration postoperative IOP and inhibit filtration postoperative scarring and neovascularization. These findings indicate its potential application in ophthalmology as an implantable drug delivery system for the treatment to ocular anterior segment diseases in the future.
